# Supplementary material for: Impact of housing conditions on changes in youth’s mental health following the initial national COVID-19 lockdown: a cohort study
Source: Sci Rep. 2022 Feb 4;12:1939. doi: 10.1038/s41598-022-04909-5 (PMC8816918; doi:10.1038/s41598-022-04909-5)

eSupplement

Table of Contents, page 1

eTables

eTable 1. Changes in mental well-being (No. = 7445), page 2

eTable 2. Onset of low mental well-being (No. = 6537), page 3

eTable 3. Changes in quality of life (No. = 7445), page 4

eTable 4. Onset of low quality of life (No. = 6283), page 5

eTable 5. Onset of loneliness (No. = 6418), page 6

eTable 6. Changes in mental well-being, quality of life and loneliness, stratified by sex (No. = 7445), page 7

eTable 7. Joint effects of housing conditions and quarantine on changes in mental well-being, quality of life and loneliness (No. = 7418), page 8

eTable 8. Changes in quality of life, stratified by psychiatric illnesses (No. = 7418), page 9

eTable 9. Changes in category of mental well-being (No. = 7445), page 10

eTable 10. Changes in category of quality of life (No. = 7445), page 11

eTable 11. Changes in category of loneliness (No. = 7445), page 12

eFigures

eFigure 1. Flow of participation, page 13

eTable 1. Changes in mental well-being (No. = 7445)

		Mean difference (95 % CI)		
	no.	**Crude Model**	**Adjusted model 1**	**Adjusted model 2**
**Direct access to outdoor spaces**				
None	*286*	-0.68 [-1.03,-0.33]	-0.72 [-1.08,-0.36]	-0.75 [-1.14,-0.36]
Common yard	*465*	0.15 [-0.13,0.44]	0.06 [-0.23,0.35]	0.03 [-0.28,0.35]
Balcony	*459*	0.17 [-0.11,0.45]	0.09 [-0.20,0.38]	0.08 [-0.23,0.39]
Multiple outdoor spaces	*1889*	0.09 [-0.07,0.25]	0.07 [-0.09,0.23]	0.06 [-0.11,0.22]
Garden	*4346*	0.00 [0.00,0.00]	0.00 [0.00,0.00]	0.00 [0.00,0.00]
**Urbanicity**				
Urban	*2698*	-0.05 [-0.21,0.10]	-0.19 [-0.36,-0.01]	-0.20 [-0.39,-0.02]
Semi-urban	*1602*	-0.08 [-0.25,0.10]	-0.13 [-0.32,0.06]	-0.13 [-0.32,0.06]
Rural	*3145*	0.00 [0.00,0.00]	0.00 [0.00,0.00]	0.00 [0.00,0.00]
**Household density**				
Above median	*3457*	-0.18 [-0.32,-0.05]	-0.19 [-0.32,-0.05]	-0.21 [-0.35,-0.07]
Below median	*3988*	0.00 [0.00,0.00]	0.00 [0.00,0.00]	0.00 [0.00,0.00]
**Household composition**				
Alone	*436*	-0.17 [-0.47,0.12]	-0.21 [-0.51,0.10]	-0.03 [-0.36,0.31]
Roomies/friends	*410*	0.12 [-0.18,0.43]	0.03 [-0.28,0.35]	0.13 [-0.22,0.47]
Partner	*835*	0.30 [0.07,0.53]	0.27 [0.03,0.50]	0.35 [0.10,0.60]
Parents and siblings/children	*2880*	0.06 [-0.09,0.21]	0.09 [-0.06,0.25]	0.09 [-0.06,0.24]
Parents only	*2884*	0.00 [0.00,0.00]	0.00 [0.00,0.00]	0.00 [0.00,0.00]

CI: Confidence Intervals.
Mean difference and 95 % CI presented.
Adjusted Model 1. Adjusted for age, sex, current education, part-time work, moving, and geographical region.
Adjusted Model 2. Adjusted model 1, additionally mutually adjusted for other housing conditions

eTable 2. Onset of low mental well-being (No. = 6537)

		OR (95% CI)		
	no.	**Crude Model**	**Adjusted model 1**	**Adjusted model 2**
**Direct access to outdoor spaces**				
None	*56/246*	1.97 [1.44,2.70]	2.29 [1.65,3.17]	1.72 [1.20,2.48]
Common yard	*75/402*	1.54 [1.18,2.01]	1.76 [1.33,2.33]	1.45 [1.06,1.98]
Balcony	*64/390*	1.32 [0.99,1.75]	1.47 [1.09,1.97]	1.24 [0.90,1.69]
Multiple outdoor spaces	*218/1657*	1.01 [0.86,1.20]	1.09 [0.92,1.30]	1.02 [0.85,1.22]
Garden	*499/3842*	1.00 [1.00,1.00]	1.00 [1.00,1.00]	1.00 [1.00,1.00]
**Urbanicity**				
Urban	*348/2392*	1.15 [0.98,1.35]	1.27 [1.06,1.53]	1.14 [0.94,1.38]
Semi-urban	*210/1394*	1.20 [1.00,1.44]	1.21 [1.00,1.48]	1.21 [1.00,1.48]
Rural	*354/2751*	1.00 [1.00,1.00]	1.00 [1.00,1.00]	1.00 [1.00,1.00]
**Household density**				
Above median	*445/3528*	1.27 [1.11,1.46]	1.28 [1.11,1.48]	1.26 [1.08,1.46]
Below median	*467/3009*	1.00 [1.00,1.00]	1.00 [1.00,1.00]	1.00 [1.00,1.00]
**Household composition**				
Alone	*79/369*	1.70 [1.29,2.23]	2.03 [1.52,2.72]	1.62 [1.17,2.23]
Roomies/friends	*64/366*	1.32 [0.98,1.77]	1.58 [1.16,2.15]	1.24 [0.88,1.74]
Partner	*106/710*	1.09 [0.86,1.38]	1.18 [0.93,1.51]	1.03 [0.79,1.34]
Parents and siblings/children	*351/2535*	0.86 [0.73,1.02]	0.84 [0.71,0.99]	0.84 [0.71,1.00]
Parents only	*312/2557*	1.00 [1.00,1.00]	1.00 [1.00,1.00]	1.00 [1.00,1.00]

OR: Odds Ratio, CI: Confidence Intervals.
OR and 95 % CI presented.
Adjusted Model 1. Adjusted for age, sex, current education, part-time work, moving, and geographical region.
Adjusted Model 2. Adjusted model 1, additionally mutually adjusted for other housing conditions
Participants with low mental well-being scores at baseline excluded (no. = 912).
Mental well-being scores ≤20 points on the SWEMWBS indicate possible or probable depression or anxiety.

eTable 3. Changes in QoL (No. = 7445)

		Mean difference (95 % CI)		
	no.	**Crude Model**	**Adjusted model 1**	**Adjusted model 2**
**Direct access to outdoor spaces**				
None	*286*	0.19 [-0.06,0.45]	0.13 [-0.13,0.39]	0.05 [-0.23,0.33]
Common yard	*464*	0.28 [0.08,0.49]	0.25 [0.04,0.46]	0.16 [-0.07,0.39]
Balcony	*459*	0.07 [-0.14,0.28]	0.04 [-0.17,0.25]	-0.04 [-0.27,0.18]
Multiple outdoor spaces	*1889*	0.21 [0.09,0.32]	0.17 [0.05,0.29]	0.14 [0.02,0.26]
Garden	*4346*	0.00 [0.00,0.00]	0.00 [0.00,0.00]	0.00 [0.00,0.00]
**Urbanicity**				
Urban	*2698*	0.03 [-0.08,0.14]	-0.04 [-0.17,0.09]	-0.09 [-0.22,0.04]
Semi-urban	*1602*	0.02 [-0.11,0.15]	-0.01 [-0.15,0.13]	-0.00 [-0.14,0.13]
Rural	*3145*	0.00 [0.00,0.00]	0.00 [0.00,0.00]	0.00 [0.00,0.00]
**Household density**				
Above median	*3457*	-0.05 [-0.15,0.04]	-0.07 [-0.17,0.03]	-0.11 [-0.21,-0.01]
Below median	*3988*	0.00 [0.00,0.00]	0.00 [0.00,0.00]	0.00 [0.00,0.00]
**Household composition**				
Alone	*436*	0.04 [-0.17,0.26]	-0.01 [-0.23,0.22]	-0.04 [-0.28,0.21]
Roomies/friends	*410*	0.08 [-0.14,0.31]	0.07 [-0.16,0.30]	0.10 [-0.15,0.35]
Partner	*835*	0.36 [0.20,0.53]	0.42 [0.24,0.59]	0.40 [0.22,0.58]
Parents and siblings/children	*2880*	-0.06 [-0.17,0.05]	-0.05 [-0.16,0.06]	-0.05 [-0.16,0.06]
Parents only	*2884*	0.00 [0.00,0.00]	0.00 [0.00,0.00]	0.00 [0.00,0.00]

QoL: Quality of Life, CI: Confidence Intervals.
Mean difference and 95 % CI.
Adjusted Model 1. Adjusted for age, sex, current education, part-time work, moving, and geographical region.
Adjusted Model 2. Adjusted model 1, additionally mutually adjusted for other housing conditions

eTable 4. Onset of low QoL (No. = 6283)

		OR (95 % CI)		
	no.	**Crude Model**	**Adjusted model 1**	**Adjusted model 2**
**Direct access to outdoor spaces**				
None	*78/218*	1.10 [0.83,1.46]	1.31 [0.97,1.76]	1.18 [0.85,1.64]
Common yard	*113/385*	0.82 [0.65,1.03]	0.91 [0.72,1.16]	0.86 [0.66,1.12]
Balcony	*131/379*	1.04 [0.83,1.30]	1.16 [0.92,1.46]	1.12 [0.87,1.43]
Multiple outdoor spaces	*437/1585*	0.75 [0.66,0.85]	0.82 [0.72,0.94]	0.81 [0.70,0.93]
Garden	*1250/3716*	1.00 [1.00,1.00]	1.00 [1.00,1.00]	1.00 [1.00,1.00]
**Urbanicity**				
Urban	*706/2273*	0.93 [0.82,1.05]	1.04 [0.90,1.20]	1.04 [0.90,1.20]
Semi-urban	*433/1345*	0.98 [0.85,1.13]	0.99 [0.85,1.15]	0.98 [0.84,1.14]
Rural	*870/2665*	1.00 [1.00,1.00]	1.00 [1.00,1.00]	1.00 [1.00,1.00]
**Household density**				
Above median	*953/2859*	1.12 [1.01,1.25]	1.17 [1.05,1.30]	1.18 [1.06,1.33]
Below median	*1056/3424*	1.00 [1.00,1.00]	1.00 [1.00,1.00]	1.00 [1.00,1.00]
**Household composition**				
Alone	*121/338*	1.17 [0.93,1.49]	1.40 [1.09,1.81]	1.38 [1.04,1.82]
Roomies/friends	*106/340*	0.95 [0.75,1.22]	1.10 [0.85,1.43]	1.08 [0.81,1.44]
Partner	*195/677*	0.85 [0.71,1.03]	0.86 [0.70,1.04]	0.86 [0.70,1.06]
Parents and siblings/children	*801/2486*	1.00 [0.89,1.13]	0.98 [0.87,1.11]	0.98 [0.87,1.11]
Parents only	*786/2442*	1.00 [1.00,1.00]	1.00 [1.00,1.00]	1.00 [1.00,1.00]

QoL: Quality of Life, OR: Odds Ratios, CI: Confidence Intervals.
OR and 95 % CI presented.
Adjusted model 1. Adjusted for age, sex, current education, part-time work, moving, and geographical region.
Adjusted model 2. Adjusted model 1, additionally mutually adjusted for other housing conditions
Participants with low quality of life at baseline excluded (no. =1162)

eTable 5. Onset of loneliness (No. = 6418)

		OR (95 % CI)		
	no. cases/no.	**Crude Model**	**Adjusted model 1**	**Adjusted model 2**
**Direct access to outdoor spaces**				
None	*64/234*	1.56 [1.16,2.11]	1.82 [1.34,2.48]	1.44 [1.01,2.04]
Common yard	*74/394*	0.96 [0.74,1.25]	1.05 [0.80,1.38]	0.98 [0.73,1.34]
Balcony	*89/378*	1.28 [1.00,1.64]	1.41 [1.09,1.83]	1.37 [1.04,1.82]
Multiple outdoor spaces	*295/1619*	0.93 [0.80,1.07]	0.99 [0.85,1.15]	0.97 [0.83,1.13]
Garden	*736/3793*	1.00 [1.00,1.00]	1.00 [1.00,1.00]	1.00 [1.00,1.00]
**Urbanicity**				
Urban	*454/2315*	1.01 [0.88,1.16]	1.04 [0.88,1.22]	1.00 [0.85,1.19]
Semi-urban	*276/1383*	1.04 [0.88,1.22]	1.04 [0.87,1.24]	1.02 [0.85,1.21]
Rural	*528/2720*	1.00 [1.00,1.00]	1.00 [1.00,1.00]	1.00 [1.00,1.00]
**Household density**				
Above median	*620/2945*	1.18 [1.05,1.34]	1.21 [1.07,1.37]	1.30 [1.14,1.48]
Below median	*638/3473*	1.00 [1.00,1.00]	1.00 [1.00,1.00]	1.00 [1.00,1.00]
**Household composition**				
Alone	*121/362*	1.94 [1.53,2.46]	2.37 [1.84,3.06]	2.12 [1.59,2.82]
Roomies/friends	*51/352*	0.65 [0.48,0.89]	0.74 [0.54,1.03]	0.66 [0.47,0.94]
Partner	*111/698*	0.73 [0.58,0.91]	0.77 [0.61,0.98]	0.72 [0.56,0.92]
Parents and siblings/children	*461/2508*	0.87 [0.76,1.00]	0.85 [0.74,0.98]	0.86 [0.74,0.99]
Parents only	*514/2498*	1.00 [1.00,1.00]	1.00 [1.00,1.00]	1.00 [1.00,1.00]

OR: Odds Ratios, CI: Confidence Intervals.
OR and 95 % CI presented.
Adjusted Model 1. Adjusted for age, sex, current education, part-time work, moving, and geographical region.
Adjusted Model 2. Adjusted model 1, additionally mutually adjusted for other housing conditions
Participants with loneliness at baseline excluded (no. = 1027)

eTable 6. Changes in mental well-being, quality of life and loneliness, stratified by sex (No.= 7445)

		Female		Male
**Mental well-being**	no.	Mean difference (95 % CI)	no.	Mean difference (95 % CI)
**Household density**				
Above median	*2397*	-0.17 [-0.34,-0.01]	*1060*	-0.27 [-0.53,-0.00]
Below median	*2816*	0.00 [0.00,0.00]	*1172*	0.00 [0.00,0.00]
**Household composition**				
Alone	*278*	0.30 [-0.11,0.71]	*158*	-0.62 [-1.20,-0.03]
Roomies/friends	*267*	0.46 [0.04,0.88]	*143*	-0.51 [-1.12,0.10]
Partner	*655*	0.43 [0.14,0.71]	*180*	0.15 [-0.37,0.68]
Parents and siblings/children	*2042*	0.22 [0.04,0.40]	*838*	-0.21 [-0.49,0.07]
Parents only	*1971*	0.00 [0.00,0.00]	*913*	0.00 [0.00,0.00]
**QoL**	no.	Mean difference (95 % CI)	*no.*	Mean difference (95 % CI)
**Household composition**				
Alone	*278*	-0.17 [-0.48,0.14]	*158*	0.22 [-0.16,0.61]
Roomies/friends	*267*	0.28 [-0.04,0.60]	*143*	-0.21 [-0.61,0.19]
Partner	*655*	0.48 [0.26,0.70]	*180*	0.09 [-0.26,0.44]
Parents and siblings/children	*2042*	-0.06 [-0.19,0.08]	*838*	-0.02 [-0.21,0.17]
Parents only	*1971*	0.00 [0.00,0.00]	*913*	0.00 [0.00,0.00]
**Loneliness**[a]	no. cases/no.	OR (95 % CI)	no. cases/no.	OR (95 % CI)
**Household composition**				
Alone	*81/221*	1.81 [1.28,2.56]	*40/141*	3.15 [1.85,5.36]
Roomies/friends	*41/224*	0.68 [0.45,1.02]	*10/128*	0.60 [0.28,1.29]
Partner	*100/534*	0.74 [0.56,0.97]	*11/164*	0.52 [0.26,1.04]
Parents and siblings/children	*344/1740*	0.74 [0.62,0.87]	*117/768*	1.40 [1.04,1.87]
Parents only	*418/1674*	1.00 [1.00,1.00]	*96/824*	1.00 [1.00,1.00]

CI: Confidence Interval, OR: Odds Ratio, QoL: Quality of Life.
Mean difference and 95 % CI and OR and 95 % CI presented.
Results displayed for interaction terms for which the likelihood ratio test indicated interaction (p<0.2).
Adjusted Model 2: Adjusted for age, sex, current education, part-time work, moving, and geographical region, and additionally mutually adjusted for housing conditions.
[a]Participants with loneliness at baseline excluded (no. = 1023)

eTable 7. Joint effects of housing conditions and quarantine on changes in mental well-being, quality of life and loneliness (No. = 7418)

		Mean difference (95 % CI)	
	no.	**Mental well-being**	**QoL**
**Direct access to outdoor spaces**			
No access, NQ	*214*	-1.01 [-1.45,-0.57]	N/A
No access, Q	*72*	-0.06 [-0.77,0.65]	N/A
Common yard, NQ	*346*	0.06 [-0.30,0.41]	N/A
Common yard, Q	*117*	-0.19 [-0.75,0.37]	N/A
Balcony, NQ	*354*	0.10 [-0.24,0.45]	N/A
Balcony, Q	*103*	-0.18 [-0.77,0.41]	N/A
Multiple outdoor spaces, NQ	*1445*	0.07 [-0.12,0.26]	N/A
Multiple outdoor spaces, Q	*439*	-0.07 [-0.36,0.23]	N/A
Garden, NQ (referent)	*3292*	0.00 [0.00,0.00]	N/A
Garden, Q	*1036*	-0.07 [-0.28,0.13]	N/A
**Urbanity**			
Urban, NQ	*2073*	N/A	-0.22 [-0.42,-0.01]
Urban, Q	*615*	N/A	-0.31 [-0.60,-0.03]
Semi-urban, NQ	*1223*	N/A	-0.17 [-0.39,0.04]
Semi-urban, Q	*373*	N/A	-0.10 [-0.43,0.23]
Rural, NQ (referent)	*2355*	N/A	0.00 [0.00,0.00]
Rural, Q	*779*	N/A	-0.12 [-0.36,0.12]
**Household composition**			
Alone, NQ	*327*	-0.05 [-0.42,0.33]	N/A
Alone, Q	*109*	0.03 [-0.56,0.62]	N/A
Roomies/friends, NQ	*298*	-0.05 [-0.44,0.35]	N/A
Roomies/friends, Q	*112*	0.60 [0.02,1.17]	N/A
Partner, NQ	*643*	0.43 [0.15,0.71]	N/A
Partner, Q	*191*	0.07 [-0.38,0.51]	N/A
Parents and siblings/children, NQ	*2171*	0.11 [-0.06,0.29]	N/A
Parents and siblings/children, Q	*696*	-0.02 [-0.27,0.23]	N/A
Parents only, NQ (referent)	*2212*	0.00 [0.00,0.00]	N/A
Parents only, Q	*659*	-0.06 [-0.32,0.20]	N/A
**Total**	7418[a]		

CI: Confidence Interval, OR: Odds Ratio, QoL: Quality of Life, Q: Quarantined, NQ: Not Quarantined, N/A: Not applicable.
Mean difference and 95 % CI and OR and 95 % CI presented.
Results displayed for interaction terms for which the likelihood ratio test indicated interaction (p<0.2), otherwise N/A is displayed
Adjusted for age, sex, current education, part-time work, moving, and geographical region, and additionally mutually adjusted for housing conditions
[a]27 observations omitted due to lacking data on quarantine status

eTable 8. Changes in QoL, stratified by psychiatric illnesses (No. = 7418)[a]

	No psychiatric illness		Psychiatric illness	
	no.	Mean difference (95 % CI)	*no.*	Mean difference (95 % CI)
**Household composition**				
Alone	*341*	-0.00 [-0.27,0.26]	*95*	0.08 [-0.66,0.83]
Roomies/friends	*350*	0.10 [-0.17,0.37]	*60*	-0.23 [-1.12,0.67]
Partner	*709*	0.43 [0.23,0.62]	*125*	0.41 [-0.26,1.08]
Parents and siblings/children	*2556*	-0.07 [-0.19,0.05]	*311*	0.15 [-0.29,0.59]
Parents only	*2494*	0.00 [0.00,0.00]	*377*	0.00 [0.00,0.00]
**Total**	*6450*		*968*	

QoL: Quality of Life, CI: Confidence Interval.
Mean difference and 95 % CI presented.
Results displayed for interaction terms for which the likelihood ratio test indicated interaction (p<0.2).
Adjusted Model 2: Adjusted for age, sex, current education, part-time work, moving, and geographical region, and additionally mutually adjusted for housing conditions
[a]27 observations omitted due to missing data on psychiatric illnesses.

eTable 9. Changes in category of mental well-being (No. = 7445)

	no. cases, (ref.normal to normal) low to low/low to normal/normal to low	RRR (95 % CI) Low to low (6.9 %)	Low to normal (5.3 %)	Normal to low (12.3 %)
**Direct access to outdoor spaces**				
None	(ref. 190)24/16/56	1.46 [0.88,2.40]	1.11 [0.62,1.99]	1.71 [1.19,2.45]
Common yard	(ref. 327)31/32/75	1.22 [0.79,1.89]	1.36 [0.87,2.13]	1.44 [1.05,1.96]
Balcony	(ref. 326)40/29/64	1.53 [1.04,2.26]	1.25 [0.80,1.94]	1.23 [0.90,1.68]
Multiple outdoor spaces	(ref. 1439)121/111/218	1.03 [0.81,1.29]	1.27 [0.99,1.63]	1.03 [0.86,1.24]
Garden	(ref. 3343)295/209/499	1.00 [1.00,1.00]	1.00 [1.00,1.00]	1.00 [1.00,1.00]
**Urbanicity**				
Urban	(ref. 2044)160/146/348	0.73 [0.56,0.94]	0.95 [0.72,1.26]	1.14 [0.94,1.38]
Semi-urban	(ref. 1184)116/92/210	0.98 [0.76,1.27]	1.13 [0.84,1.50]	1.22 [1.00,1.49]
Rural	(ref. 2397)235/159/354	1.00 [1.00,1.00]	1.00 [1.00,1.00]	1.00 [1.00,1.00]
**Household density**				
Above median	(ref. 2542)267/181/467	1.37 [1.14,1.66]	1.01 [0.82,1.26]	1.26 [1.09,1.46]
Below median	(ref. 3083)244/216/445	1.00 [1.00,1.00]	1.00 [1.00,1.00]	1.00 [1.00,1.00]
**Household composition**				
Alone	(ref. 290)35/32/79	1.48 [0.96,2.29]	1.44 [0.91,2.28]	1.58 [1.15,2.19]
Roomies/friends	(ref. 302)25/19/64	1.02 [0.62,1.68]	0.85 [0.49,1.46]	1.23 [0.87,1.73]
Partner	(ref. 604)64/61/106	1.28 [0.92,1.78]	1.28 [0.90,1.82]	1.02 [0.78,1.33]
Parents and siblings/children	(ref. 2245)194/129/312	0.97 [0.79,1.20]	0.81 [0.64,1.04]	0.84 [0.71,0.99]
Parents only	(ref. 2184)193/156/351	1.00 [1.00,1.00]	1.00 [1.00,1.00]	1.00 [1.00,1.00]
**Total (no.)**	(ref.5625)511/397/912			

RRR: Relative Risk Ratios, CI: Confidence Intervals.
RRR and 95 % CI presented.
Adjusted Model 2: Adjusted for age, sex, current education, part-time work, moving, geographical region, and mutual adjustment for housing conditions.

eTable 10. Changes in category of QoL (No. = 7445)

	no. cases, (ref.normal to normal) low to low/low to normal/normal to low	RRR (95 % CI) Low to low (9.5 %)	Low to normal (6.1 %)	Normal to low (27.1 %)
**Direct access to outdoor spaces**				
None	(ref. 140)37/31/78	1.41 [0.91,2.18]	1.87 [1.17,3.00]	1.16 [0.84,1.60]
Common yard	(ref. 272)45/35/113	0.95 [0.65,1.38]	1.11 [0.72,1.71]	0.86 [0.66,1.12]
Balcony	(ref. 248)46/34/131	1.09 [0.76,1.58]	1.21 [0.80,1.85]	1.11 [0.87,1.42]
Multiple outdoor spaces	(ref. 1148)189/115/437	1.06 [0.87,1.30]	1.03 [0.81,1.32]	0.81 [0.71,0.93]
Garden	(ref. 2466)395/235/1250	1.00 [1.00,1.00]	1.00 [1.00,1.00]	1.00 [1.00,1.00]
**Urbanicity**				
Urban	(ref. 1567)253/172/706	0.88 [0.71,1.10]	1.05 [0.80,1.37]	1.04 [0.90,1.21]
Semi-urban	(ref. 912)158/99/433	0.98 [0.78,1.23]	1.10 [0.83,1.44]	0.99 [0.85,1.15]
Rural	(ref. 1795)301/179/870	1.00 [1.00,1.00]	1.00 [1.00,1.00]	1.00 [1.00,1.00]
**Household density**				
Above median	(ref. 1906)378/220/953	1.51 [1.28,1.79]	1.12 [0.91,1.37]	1.18 [1.06,1.32]
Below median	(ref. 2368)334/230/1056	1.00 [1.00,1.00]	1.00 [1.00,1.00]	1.00 [1.00,1.00]
**Household composition**				
Alone	(ref. 217)64/34/121	2.14 [1.49,3.08]	1.49 [0.95,2.35]	1.39 [1.06,1.83]
Roomies/friends	(ref. 234)40/30/106	1.15 [0.76,1.73]	1.38 [0.87,2.20]	1.05 [0.79,1.40]
Partner	(ref. 482)81/77/195	1.05 [0.78,1.42]	1.54 [1.11,2.14]	0.84 [0.68,1.04]
Parents and siblings/children	(ref. 1685)246/148/801	0.84 [0.70,1.01]	0.90 [0.72,1.14]	0.98 [0.87,1.11]
Parents only	(ref. 1656)281/161/786	1.00 [1.00,1.00]	1.00 [1.00,1.00]	1.00 [1.00,1.00]
**Total (no.)**	(ref. 4274)712/450/2009			

QoL: Quality of Life, RRR: Relative Risk Ratios, CI: Confidence Intervals.
RRR and 95 % CI presented.
Adjusted model 2: Adjusted for age, sex, current education, part-time work, moving, geographical region, and mutual adjustment for housing conditions.

eTable 11. Changes in category of loneliness (No. = 7445)

	no. cases, (ref.not lonely to not lonely) lonely to lonely/lonely to not lonely/not lonely to lonely	RRR (95 % CI) Lonely to lonely (16.9 %)	Lonely to not lonely (7.6 %)	Not lonely to lonely (6.1 %)
**Direct access to outdoor spaces**				
None	(ref. 170)26/26/64	1.91 [1.16,3.16]	1.41 [0.87,2.27]	1.44 [1.02,2.03]
Common yard	(ref. 320)39/32/74	1.63 [1.08,2.48]	0.94 [0.62,1.44]	0.98 [0.73,1.33]
Balcony	(ref. 289)28/53/89	1.31 [0.84,2.04]	1.75 [1.23,2.48]	1.35 [1.02,1.78]
Multiple outdoor spaces	(ref. 1324)115/155/295	1.15 [0.90,1.46]	1.21 [0.98,1.51]	0.97 [0.83,1.13]
Garden	(ref. 3057)255/298/736	1.00 [1.00,1.00]	1.00 [1.00,1.00]	1.00 [1.00,1.00]
**Urbanicity**				
Urban	(ref. 1861)169/214/454	1.00 [0.77,1.30]	0.96 [0.76,1.23]	1.01 [0.85,1.20]
Semi-urban	(ref.1107)99/120/276	1.01 [0.77,1.33]	0.98 [0.76,1.26]	1.02 [0.85,1.21]
Rural	(ref.2192)195/230/528	1.00 [1.00,1.00]	1.00 [1.00,1.00]	1.00 [1.00,1.00]
**Household density**				
Above median	(ref. 2325)225/287/620	1.19 [0.98,1.46]	1.23 [1.03,1.48]	1.29 [1.13,1.46]
Below median	(ref. 2835)238/277/638	1.00 [1.00,1.00]	1.00 [1.00,1.00]	1.00 [1.00,1.00]
**Household composition**				
Alone	(ref. 241)43/31/121	1.88 [1.23,2.89]	1.23 [0.79,1.92]	2.10 [1.58,2.79]
Roomies/friends	(ref. 301)29/29/51	1.01 [0.63,1.61]	0.83 [0.52,1.31]	0.66 [0.47,0.94]
Partner	(ref. 587)38/99/111	0.63 [0.42,0.94]	1.48 [1.11,1.97]	0.72 [0.56,0.92]
Parents and siblings/children	(ref. 2047)179/193/461	0.99 [0.79,1.23]	0.89 [0.72,1.09]	0.86 [0.74,0.99]
Parents only	(ref. 1984)174/212//514	1.00 [1.00,1.00]	1.00 [1.00,1.00]	1.00 [1.00,1.00]
**Total (no.)**	(ref. 5160)463/564/1258			

RRR: Relative Risk Ratios, CI: Confidence Intervals.
RRR and 95 % CI presented.
Adjusted model 2: Adjusted for age, sex, current education, part-time work, moving, geographical region, and mutual adjustment for housing conditions.

eFigure 1. Flow of participation

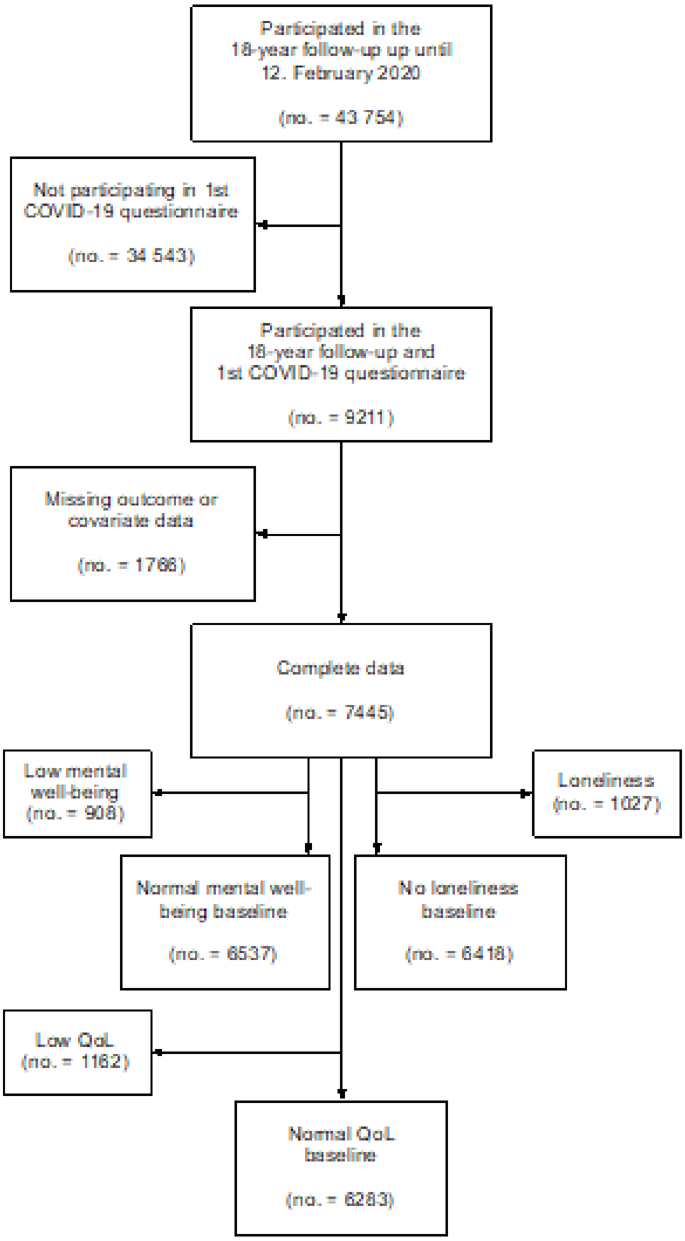

Supplement: Supplementary file 1 — Supplementary Information. [file 41598_2022_4909_MOESM1_ESM.pdf]
